# Delay Tracking of Spread-Spectrum Signals for Indoor Optical Ranging

**DOI:** 10.3390/s141223176

**Published:** 2014-12-05

**Authors:** David Salido-Monzú, Ernesto Martín-Gorostiza, José Luis Lázaro-Galilea, Eduardo Martos-Naya, Andreas Wieser

**Affiliations:** 1 Department of Electronics, University of Alcalá, Madrid 28871, Spain; E-Mails: ernesto@depeca.uah.es (E.M.-G.); lazaro@depeca.uah.es (J.L.L.-G.); 2 Communication Engineering Department, University of Málaga, Campus de Teatinos, Málaga 29010, Spain; E-Mail: eduardo@ic.uma.es; 3 Institute of Geodesy and Photogrammetry, ETH Zürich, Zürich 8093, Switzerland; E-Mail: andreas.wieser@geod.baug.ethz.ch

**Keywords:** optical ranging, spread-spectrum, delay-locked loop, multipath mitigation, indoor positioning

## Abstract

Delay tracking of spread-spectrum signals is widely used for ranging in radio frequency based navigation. Its use in non-coherent optical ranging, however, has not been extensively studied since optical channels are less subject to narrowband interference situations where these techniques become more useful. In this work, an early-late delay-locked loop adapted to indoor optical ranging is presented and analyzed. The specific constraints of free-space infrared channels in this context substantially differ from those typically considered in radio frequency applications. The tracking stage is part of an infrared differential range measuring system with application to mobile target indoor localization. Spread-spectrum signals are used in this context to provide accurate ranging while reducing the effect of multipath interferences. The performance of the stage regarding noise and dynamic errors is analyzed and validated, providing expressions that allow an adequate selection of the design parameters depending on the expected input signal characteristics. The behavior of the stage in a general multipath scenario is also addressed to estimate the multipath error bounds. The results, evaluated under realistic conditions corresponding to an 870 nm link with 25 MHz chip-rate, built with low-cost up-to-date devices, show that an overall error below 6% of a chip time can be achieved.

## 1. Introduction

Indoor positioning systems have evolved in the last two decades inspired by global navigation satellite systems (GNSS), and at the same time suffering from their own problems, due to the specific indoor characteristics. In contrast with GNSS, which is the accepted technology for global positioning outdoors, there is not one single solution, nor a unique technology, universally accepted as best solution for indoor localization. Hence, many different systems have been proposed during recent years, based on computer vision, radio signals, ultrasound, optical signals and, more recently, a large number of new approaches based on inertial measurement units (IMU) and radio frequency (RF) communications networks such as GSM or WLAN [1,2].

Regardless of the technology used, the most common problems that indoor systems must deal with are related to signal quality, positioning-anchors deployment strategy (antennas, receivers, emitters, *etc.*), non-line-of-sight situations, dynamic localization, interferences from other devices, and, like with GNSS, multipath contributions [3]. Among these, the latter may cause a major contribution to the error in many typical indoor environments, mainly with those technologies designed for accurate applications, and it is nowadays one of the most difficult handicaps to overcome.

Non-coherent optical ranging is usually carried out by time-of-flight measurements on pulsed signals [4] or phase-shift measurements on continuous wave modulated signals [5]. Phase-based techniques do not inherently provide any multipath mitigation and pulsed systems, usually implemented with laser, require very high bandwidth to discriminate multipath components close to the direct path.

Most of the solutions proposed for the problem of multipath in the last two decades have been developed in the context of GNSS. The classical methods are based on special correlator design [6] while, in more recent approaches, multipath estimation methods [7] are used. The latter are based on the original concept of the Multipath Estimating Delay-Locked Loop [8,9], evolved into different improved variants. These techniques provide higher multipath rejection than classical ones but require long integration times and high sampling rates, being limited to static applications with more complex receivers. The classical approach is generally based on the features of the correlation or discrimination functions. The effect of the multipath contributions can be mitigated by proper design of the correlators used in these architectures, like narrow correlators [10], strobe and edge correlators [11], gated correlators [12] or high resolution correlators [13]. These techniques can mitigate non-severe multipath effects to a great extent when applied in high performance receivers, however, their effectiveness is highly reduced in low cost systems providing smaller bandwidth and sampling rate.

Unfortunately, none of these mitigation techniques is directly applicable to the proposed optical application. The main limitation in the design of the ranging architecture is determined by the strong trade-off between SNR, optical devices angle and channel bandwidth to meet the precision, coverage and dynamic response requirements of the applications [5]. Due to this trade-off, the achievable channel bandwidth using low-cost up-to-date devices is on the order of tens of MHz, limiting the possible ranging techniques applied to the system. Time discrimination methods, such as those used in pulsed laser ranging, require much higher bandwidths to resolve indoor multipath, while correlator-based methods would provide very limited mitigation when applied to the available bandwidth using affordable digitization systems.

On the other hand, some very specific interferometric applications use direct-sequence spread-spectrum (DSSS) modulations on the sinusoidal heterodyne signals to discriminate different paths based on their delays [14], making use of selective coherent demodulation for each path [15,16]. The selective demodulation requires information of the approximated delay for each path. In these systems, these approximations are already known and very small displacements (in the order of pm) are measured. Although the initial delay is not known in the proposed system, this coherent demodulation approach could be used by previously estimating the delay of the DSSS-modulated signal. This delay estimation, given the DSSS signals characteristics, can be achieved by means of a delay-locked loop (DLL) architecture, adapted from those typically used in GNSS.

Thus, the method proposed in this paper is inspired by both technologies, applying a multipath mitigation solution derived from interferometric applications while solving the initial delay estimation using RF synchronization techniques, where the narrow correlator concept is also applied for improved multipath rejection.

This paper is particularly focused on the performance study of the tracking stage used to obtain the delay estimation of the received spread-spectrum signal. Spread-spectrum signals applied to ranging are widely used in RF, mainly for GNSS, and, to a lesser extent, ultrasound systems [17]; however, its application to optical systems has not been extensively studied except for some very specific applications like inter-spacecraft ranging [18], using high performance lasers, or low-accuracy inter-vehicle ranging [19], focused on its multiplexing capabilities. Its application for accurate indoor optical ranging is a novel approach that requires a specific study. The tracking stage is particularized for a feasible optical link in the context of indoor localization, which causes some specific issues different to those associated with the corresponding approach in RF systems like GNSS. The goals and innovative contributions of this paper are to define the tracking stage, study analytically how it is affected by the dominant error sources, noise and dynamic effects, and to analyze the performance of the tracking stage in terms of accuracy.

A deep study of multipath effects lies outside the scope of this paper. However, a realistic multipath scenario is being addressed, providing some results on the expected multipath error in order to bound its effect on the system performance. The effect of multipath is often studied through fading models that describe the spatial and temporal variations in the attenuation of the received signal. The system proposed herein does not use signal power information for ranging. Fading does therefore hardly affect system performance, and a study of its potential impact, e.g., on delay measurement noise level, is left for future investigations. A model of the received signal is used instead, resulting from the composition of direct and multipath components reaching a receiver, where the power and delay of each component are calculated using radiometric and geometric assumptions.

The paper is organized as follows: first an overview of previous research on IR indoor positioning and a general description of the proposed ranging architecture are provided in Section 2. In Section 3, the structure and functioning of the delay tracking system are described, and the involved signals and main design trade-offs are explained. Section 4 describes the main error sources affecting the tracking system, and the expressions of the delay estimation errors are derived. Results obtained from a digital implementation of the tracking stage, with emulated received signals defined by a practical indoor situation considering up-to-date optical devices, are shown in Section 5, and compared with the theoretically expected results for validation. Some preliminary results of the estimated multipath error under realistic conditions are also provided in this section. Finally, the conclusions of this study and future works are detailed in Section 6.

## 2. Context

Previous research on IR-based indoor positioning was presented in [5]. The position of the target was calculated by hyperbolic trilateration from distance difference estimations between the target and pairs of reference points placed in the ceiling of the environment. The ranging system providing these distance differences was based on phase difference of arrival (PDOA) estimations of a sinusoidally intensity modulated near-infrared (IR) signal emitted from the target, reaching any pair of receivers placed in the aforementioned reference points. Good performance was demonstrated with this ranging system using standard up-to-date devices (IR-LED and Si PiN photodiodes), yielding positioning errors below 2.5 cm with tens of ms update-rates. However, multipath effects were identified as a critical source of error, affecting the position error at the cm to dm level depending on the environment geometry and reflective properties. This motivated the study of a new IR-based ranging scheme adapted to indoor positioning applications, that allows accurate optical ranging by reducing multipath-related errors.

**Figure 1.**
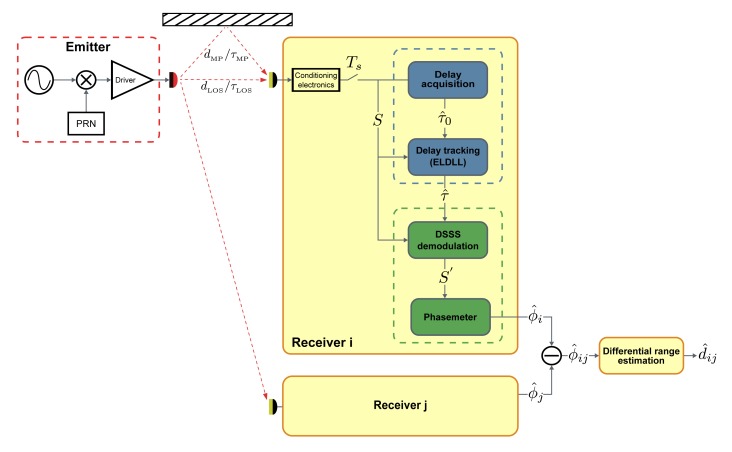
Full diagram of the ranging system.

The general structure and functioning of the proposal that contextualizes this work was presented in [20]. Figure 1 shows the structure of the full ranging system. The emitted signal intensity modulating the optical carrier is a sine tone modulated by a pseudo-random noise (PRN) spreading sequence. This process can be understood as a DSSS modulation of the sinusoidal signal. The spread-spectrum signal (*S*) reaching every receiver is tracked by a synchronization stage, and the tracked delay (*τ̂*) is used to generate a local in-phase replica of the received PRN. This local replica is used to demodulate the received signal, despreading part of its power into the original sine frequency. An I/Q-based phase estimation over the demodulated signal (*S′*), after subtraction with an equivalent one coming from another receiver, provides the final phase difference to be converted into a range difference estimation (*d̂_ij_*). Emission-reception synchronism is irrelevant as long as the final estimates are computed from measurement differences.

Multipath mitigation takes place in the DSSS demodulation process. If the tracked delay used to generate the local code replica for demodulation is closer to the line-of-sight (LOS) component than to any of the non-line-of-sight (NLOS) components, a more coherent demodulation will be applied to it. The amount of despread power in the demodulated signal *S′* depends on this coherence, therefore, a higher amount of power would be recover in the original sinusoidal frequency for the LOS component than for the rest of the signals reaching the receiver. The multipath error in the phase estimation on the demodulated signal would be reduced due to the relative power enhancement of the LOS component.

The relative delay tracked by the synchronization stage is affected by both LOS and multipath signals. This composed delay depends on the relative powers and delays of the LOS and NLOS components of the received signal. Typical indoor optical multipath situations present a highly dominant LOS component in terms of relative power. The estimated delay affected by multipath is, except for severe multipath situations, closer to the LOS delay true-value than to the delay of the faster multipath component. Therefore, in most situations, certain level of multipath power reduction can be expected. On the other hand, once the environmental conditions that define a particular multipath situation are favorable, the performance of the tracking stage affected by the dominant error sources, such as signal noise and varying delays, is crucial for the whole system.

This paper is centered on the performance study of this tracking stage providing the relative delay estimation *τ̂*.

## 3. Tracking System Description

The delay tracking stage function is to provide a continuous estimation of the relative delay of the incoming signal. This delay estimation is used to generate the local replica of the expected PRN sequence for DSSS demodulation.

The attainable accuracy in the tracking stage is essentially affected by noise, dynamic errors and multipath effects, whose impact is analyzed in Section 4.

The delay tracking is based on a baseband early-late delay-locked loop (ELDLL). This kind of tracking loop, once locked, correlates the incoming signal with slightly advanced and delayed versions of the local replica to obtain information about the alignment error with the incoming signal. This information is used to correct the local phase in order to keep lock and provide a continuous delay estimation.

The tracking stage has to be initialized in a locked state by receiving an initial delay estimation (*τ̂*_0_) whose error is within the lock-range of the loop. This estimation is provided by a coarse acquisition stage based on sweeping possible delay candidates.

**Figure 2.**
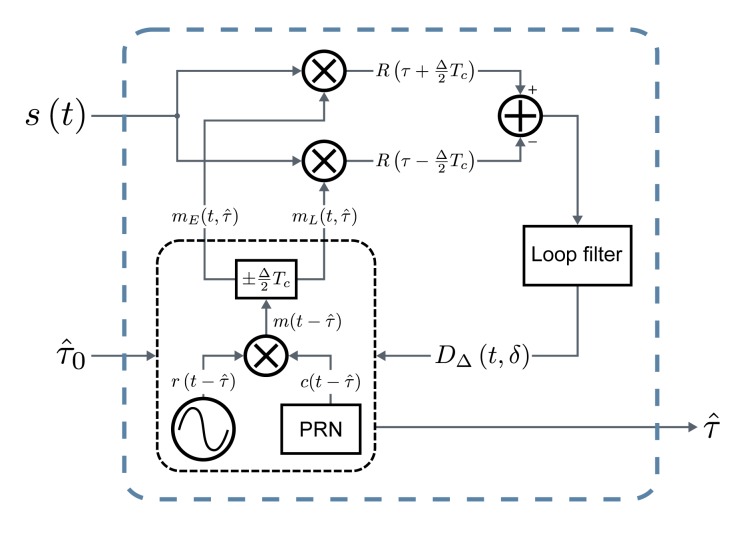
Diagram of the tracking stage (ELDLL).

The structure of the tracking stage is shown in Figure 2. Once the coarse delay estimation *τ̂*_0_ is available, it is used to initialize the local replicas generator. Early and late versions, *m_E_*(*t*, *τ̂*) and *m_L_* (*t, τ̂*), of the expected received signal separated by Δ chips, are output with an initial phase defined by *τ̂*_0_, which, after closing the loop, will be replaced by the actually tracked delay *τ̂*. These local references are correlated with the received signal *s*(*t*). The resulting early and late correlations are subtracted and filtered to obtain the discriminator output *D*_Δ_(*t, δ*), with *δ* being the normalized tracking error. Given the high symmetrical properties of the calculated correlations, the discriminator output tends to zero when the error between the delay of the incoming signal and the estimated delay *τ̂* is zero, taking negative or positive values depending on the sign of the error. The discriminator can be used as an error signal for the estimation, that can drive a feedback loop aiming at continuously driving the tracking error to 0. The discriminator output drives the local replicas generator by correcting the delay applied to the generated signals. The loop filter applied to the early-late subtraction defines the final estimation bandwidth, hence the amount of error caused by noise and varying delays, by limiting the loop bandwidth as much as the system dynamics allow it.

### 3.1. Signal Structure

The signal that modulates the intensity of the IR carrier is a sinusoidal tone modulated with a spreading sequence by DSSS. The emitted signal with power *P_tx_*, omitting the optical carrier, is

(1) $$m\left(t\right)=\sqrt{2P_{tx}}\sin\left(2\pi f_{r}t+\phi_{0}\right)\sum_{n=0}^{\infty }\sum_{k=0}^{N-1}C_{k}\prod \left(\frac{t-kT_{c}-nT_{f}}{T_{c}}\right)$$

where the sine tone is defined by its frequency *f_r_* and constant phase *ϕ*_0_ in relation to the spreading sequence phase. The spreading sequence is a non-return-to-zero (NRZ) PRN sequence periodically repeated with a frame period *T_f_* = *NT_c_* where *N* is the number of chips of the particular PRN sequence, *f_c_* = 1/*T_c_* is its chip-rate, *C_k_* ∊ {±1} is every individual chip value and

(2) $$\prod \left(\frac{t}{T_{c}}\right)=\{\begin{array}{ll} 1 & \text{if}0\leq t\leq T_{c}\\ 0 & \text{else} \end{array}$$

The signal definition is similar to typical spread-spectrum ranging and communication schemes without the inclusion of the data signal. Note that, in this optical application, however, the sine signal does not represent the RF carrier but a secondary modulation, like mentioned in [21], that enables a higher level of multipath rejection than using only the PRN signal.

The design parameters in the definition of the signal structure are the sine frequency *f_r_* and chip-rate *f_c_*, and the phase relation between them, determined by *ϕ*_0_. These values have been defined as a function of the IR link bandwidth, aiming at maximizing the precision achieved by both the tracking stage and the final distance estimation based on the phasemeter. To maximize the performance of both stages two considerations should be taken into account. In the first place, maximizing the use of the available bandwidth in terms of power is directly related with the SNR of the received and demodulated signals, hence the precision of both estimations. Secondly, the chip-rate is directly related with the precision of the tracking stage, and the sine frequency is directly related with the precision of the phase estimation; therefore, both frequencies should also be as high as possible.

Taking these considerations into account, the selected signal structure as a function of the IR channel bandwidth (*BW_IR_*) is defined as follows:

- Sine and code frequencies: both chip-rate and sine frequency are set equal to half the channel bandwidth $\left(f_{c}=f_{r}=\frac{BW_{IR}}{2}\right)$, this is, every chip modulates a full cycle of the sine signal. This way, the main lobe of the sync-shaped spread signal spectrum lies, approximately but for some distortion, centered in the channel bandwidth, containing more than 90% of the ideal (non-bandlimitted) signal power. Making both frequencies larger would increase precision under similar SNR conditions, but reduce SNR while adding higher distortion, since more power of the original signal surpasses the channel bandwidth. This distortion could be estimated and compensated up to certain level by proper pulse shaping. However, since the aim of this work is analyzing the general feasibility of the proposal, the simplest approach has been chosen.
- Sine-to-code phase: the phase relation between the sine component and the spreading sequence should be chosen to minimize distortion by the band-limited channel in the phase transitions. A zero phase in the sine modulation has been chosen (*ϕ*_0_ = 0), so that the phase shifts caused by the PRN are applied in the zero crossing points of the sine signal.
- Code type: a simple approach has been selected for this general feasibility study, using 255 chips maximum length sequences (MLS) to modulate the sine signal. The optimization of the applied PRN for this particular application will be carried out in future works.

The tracking stage functioning depends on the correlation properties of the received signal. The autocorrelation function and the discriminator function are calculated next to be used further in the document, being crucial for the performance of the tracking stage.

The phase relationship *ϕ*_0_ between the sine signal and the PRN sequence is constant. Assuming that this phase is random and uniform in [0, *T_c_*], and that the PRN has a discrete random phase uniform in [0, *N* − 1], both random variables being independent, the defined signal can be considered stationary and its autocorrelation function can be written as

(3) $$R_{m}\left(\tau \right)=\sum_{n=-\infty }^{\infty }R_{c}\left(n\right)R_{q}\left(\tau -nT_{c}\right)$$

where *R_c_*(*n*) is the autocorrelation function of the PRN sequence *C_k_*

(4) $$R_{c}\left(n\right)=\{\begin{array}{cc} 1 & \text{if}\;n=0\\ -\frac{1}{N} & \text{if}\;n\neq 0 \end{array}$$

and *R_q_* (*τ*) is the symbol autocorrelation. This symbol, including the sine signal in its definition, is

(5) $$q\left(t\right)=\prod \left(\frac{t}{T_{c}}\right)\sin\left(\frac{2\pi t}{T_{c}}\right)$$

and its autocorrelation is

(6) $$R_{q}\left(\tau \right)=\{\begin{array}{ll} \frac{1}{2}\left(1-\frac{\left|\tau \right|}{T_{c}}\right)\cos\left(\frac{2\pi \tau }{T_{c}}\right)+\frac{1}{2\pi }\sin\left(\frac{2\pi \left|\tau \right|}{T_{c}}\right) & \text{if}\left|\tau \right|<T_{c}\\ 0 & \text{if}\left|\tau \right|\geq T_{c} \end{array}$$

**Figure 3.**
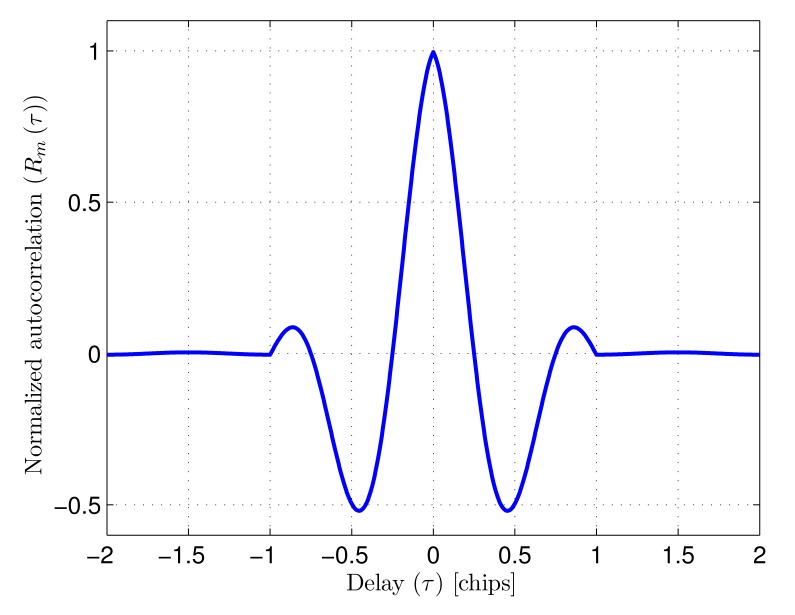
Normalized autocorrelation function *R_m_*(*τ*) of the signal *m*(*t*).

The calculated autocorrelation is depicted in Figure 3.

**Figure 4.**
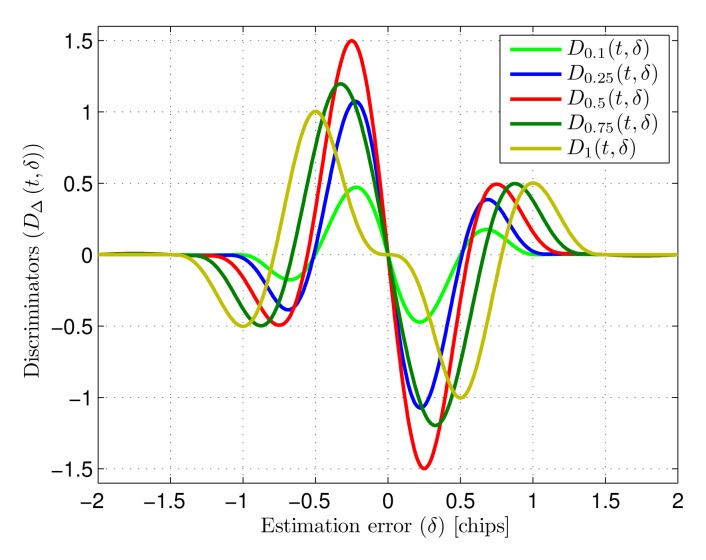
Discriminators *D*_Δ_(*t, δ*) for ideal input (multipath-free, noise-free) for different early-late spacing (Δ).

The discriminator is formed by the filtered subtraction of the early and late multipliers outputs. Considering a multipath-free and noise-free input, and assuming that the involved signals are ergodic, *i.e.*, the time average of the loop filter equals the statistical one, the normalized discriminator output can be approximated using the subtraction of the early and late autocorrelations

(7) $$D_{\Delta }\left(t,\delta \right)=R_{m}\left(\delta T_{c}+\frac{\Delta }{2}T_{c}\right)-R_{m}\left(\delta T_{c}-\frac{\Delta }{2}T_{c}\right)$$

where *δ* is the estimation error (*τ*_LOS_ − *τ̂*) normalized to *T_c_*. The discriminator shape is strongly dependent on the early-late spacing Δ. Figure 4 shows various discriminators for different early-late spacings between 0.1 chips and 1 chip. The selection of an adequate spacing is addressed in the next section.

### 3.2. Design Trade-Offs

The tracking stage has two main design trade-offs: the spacing (Δ) between the early and late replicas and the loop bandwidth (*W_L_*).

- Early-late spacing (Δ): The early-late spacing is the delay in relation to the estimated delay *τ̂* used to generate the local references. Early and late references are generated as Δ/2 chips advanced and delayed versions of expected received signal *m*(*t* − *τ̂*). This spacing, as can be seen in Figure 4, strongly affects the shape of the discriminator function, which has an influence on the effects of noise and multipath within the tracking. Different discriminator shapes provide different sensibilities in the tracking loop, as well as different amounts of transfered noise power. In addition, narrow spacings in GNSS receivers with similar architectures have been demonstrated to reduce the effect of multipath components in the estimation [10,22–24]. The selection of an adequate spacing considering these trade-offs is addressed in the next section.
- Loop bandwidth (*W_L_*): The loop filter sets the bandwidth limitation applied to the discriminator output that drives the tracking loop, ultimately defining the delay estimation final bandwidth. Small loop bandwidths increase noise filtering, yielding higher precision in static conditions. However, the ability of the loop to follow variations of the delay of the incoming signal will be poorer for small bandwidths, yielding higher dynamic errors caused by movement of the target or frequency errors due to the asynchronous emitter-receiver signal generation. The optimization of the loop bandwidth is also addressed in the next section.

## 4. Tracking System Analysis

The goal of this work is studying the general performance of the delay tracking system affected by its dominant error contributions: noise and varying delay in the input signal, related with the design trade-offs commented above. The analysis is divided into three error indicators:

- Tracking jitter $\left(\sigma_{\hat{\tau }}^{2}\right)$: the variance of the estimated delay in the presence of additive white Gaussian noise (AWGN) is first analyzed, concluding on the resulting jitter as a function of the early-late spacing, input SNR, loop bandwidth and chip-rate. The resulting jitter, together with some considerations related to multipath rejection and hardware implementation issues, leads to the selection of an adequate early-late spacing.
- Dynamic error (*∈_τ̂_*): the tracking error when the delay of the input signal varies due to target displacement and emitter-receiver asynchronism is analyzed next, concluding on the dynamic error as a function of the delay rate of change and loop bandwidth.
- Total error (*∈_T_*): defined as the sum of the typical error corresponding with the tracking jitter and the dynamic error
  (8) $$\epsilon_{T}=\sigma_{\hat{\tau }}+\in_{\hat{\tau }}$$
  The joint contribution of both error sources, linked by the loop bandwidth *W_L_*, is studied, leading to its optimization to minimize the total error of the stage under certain SNR and delay rate of change conditions.

Finally, the expression of the discriminator function in the presence of multipath is presented, and used to calculate the expected multipath error under some general NLOS conditions. The goal of this section is to bound the expected multipath effect and to define the general scenario for which a deeper multipath study will be carried out later, not analyzing the multipath error under realistic conditions.

### 4.1. Tracking Jitter

The study of the effect of noise on the tracking jitter has been carried out following standard baseband DLL analysis [25,26]. These analyses are usually carried out for spread-spectrum ranging and synchronization of communication systems, where the typical tracked signal is a PRN sequence. In this case, the analysis has been adapted to the discriminator *D*_Δ_ (*t, δ*) formed by the signal defined for the proposed ranging architecture.

The discriminator output in the presence of AWGN is explicitly analyzed here for the proposed signal structure. Once it is adequately characterized, the resulting tracking jitter yielded by linear loop analysis of the baseband DLL is applied considering the particular discriminator characteristics.

Considering a multipath-free received signal with delay *τ*_LOS_ and power *P_r_* plus an AWGN contribution *n*(*t*) having two-sided power spectral density *N*_0_/2. The output of the discriminator *D*(*t*, *τ*_LOS_, *τ̂*), expressed as a function of time and the true and estimated delays in static conditions, is

(9) $$D\left(t,\tau_{\text{LOS}},\hat{\tau }\right)=\sqrt{P_{r}}D_{\Delta }\left(t,\delta \right)+n_{f}\left(t\right)$$

where *D*_Δ_ (*t, δ*) is the ideal discriminator defined in Equation (7), and *n_f_* (*t*) is the noise contribution after the loop filter.

The jitter power caused by *n_f_* (*t*) can be calculated from the noise power spectral density before the loop filter integrated in the loop bandwidth *W_L_*. The time expression of noise before the loop filter is

(10) $$n^{\prime }\left(t,\Delta \right)=n\left(t\right)\left[m\left(t-\hat{\tau }+\frac{\Delta }{2}T_{c}\right)-m\left(t-\hat{\tau }-\frac{\Delta }{2}T_{c}\right)\right]$$

The power spectrum of *n′* (*t*) is calculated from its autocorrelation function. Considering *τ̂* as a random variable, *n′* (*t*) can be considered stationary and

(11) $$R_{n^{\prime }}\left(\tau ,\Delta \right)=E\left\{n\left(t\right)n\left(t+\tau \right)\left[m\left(t-\hat{\tau }+\frac{\Delta }{2}T_{c}\right)-m\left(t-\hat{\tau }-\frac{\Delta }{2}T_{c}\right)\right]\times \left[m\left(t+\tau -\hat{\tau }+\frac{\Delta }{2}T_{c}\right)-m\left(t+\tau -\hat{\tau }-\frac{\Delta }{2}T_{c}\right)\right]\right\}$$

Since *n*(*t*) is independent from the expected signal *m*(*t*), the expected value can be factored as

(12) $$R_{n^{\prime }}\left(\tau ,\Delta \right)=E\left[n\left(t\right)n\left(t+\tau \right)\right]E\left\{\left[m\left(t-\hat{\tau }+\frac{\Delta }{2}T_{c}\right)-m\left(t-\hat{\tau }-\frac{\Delta }{2}T_{c}\right)\right]\times \left[m\left(t+\tau -\hat{\tau }+\frac{\Delta }{2}T_{c}\right)-m\left(t+\tau -\hat{\tau }-\frac{\Delta }{2}T_{c}\right)\right]\right\}$$

The autocorrelation function of the input noise is a delta function defined by its power spectral density as

(13) $$E\left[n\left(t\right)n\left(t+\tau \right)\right]=\frac{N_{0}}{2}\delta \left(\tau \right)$$

being zero for any *τ* ≠ 0, so that

(14) $$R_{n\prime }\left(\tau ,\Delta \right)=\frac{N_{0}}{2}\delta \left(\tau \right)\left[2R_{m}\left(0\right)-2R_{m}\left(\Delta T_{c}\right)\right]$$

The two-sided power spectral density of *n′* (*t*, Δ) is the Fourier transform of its autocorrelation, being

(15) $$S_{n\prime }\left(f,\Delta \right)=\frac{N_{0}}{2}\left[2R_{m}\left(0\right)-2R_{m}\left(\Delta T_{c}\right)\right]$$

Once the effect of noise in the discriminator is properly characterized, the tracking jitter obtained by linear baseband DLL analysis, easily found in spread-spectrum literature [25,26], is applied considering the calculated noise power spectral density. The variance of the normalized estimation error is

(16) $$\sigma_{\delta }^{2}=\frac{S_{n\prime }\left(f,\Delta \right)W_{L}}{2K_{d}^{2}}$$

where *W_L_* is the two-sided closed-loop bandwidth defined by the loop filter and *K_d_* is the discriminator gain, defined by the signal power and the sensitivity of the discriminator

(17) $$K_{d}=\sqrt{\frac{P_{r}}{2}}{\frac{\partial D_{\Delta }\left(t,\delta \right)}{\partial \delta }|}_{\delta =0}$$

This analysis is valid for small estimation errors, when *δ* is around zero, assuming the discriminator has a linear behavior in that range, approximated to its slope in the origin.

Finally, undoing the normalization to the chip duration *T_c_*, the tracking variance is

(18) $$\sigma_{\hat{\tau }}^{2}=T_{c}^{2}\frac{S_{n^{\prime }}\left(f,\Delta \right)W_{L}}{P_{r}{\left({\frac{\partial D_{\Delta }\left(t,\delta \right)}{\partial \delta }|}_{\delta =0}\right)}^{2}}$$

Both the noise power spectrum *S_n_′* (*f*, Δ) and the sensitivity of the discriminator ${\frac{\partial D_{\Delta }\left(t,\delta \right)}{\partial \delta }|}_{\delta =0}$depend on the chosen early-late spacing Δ. Small spacings reduce noise transfer due to the higher correlation between noise components in the early and late branches, penalizing, however, the sensitivity of the discriminator.

**Figure 5.**
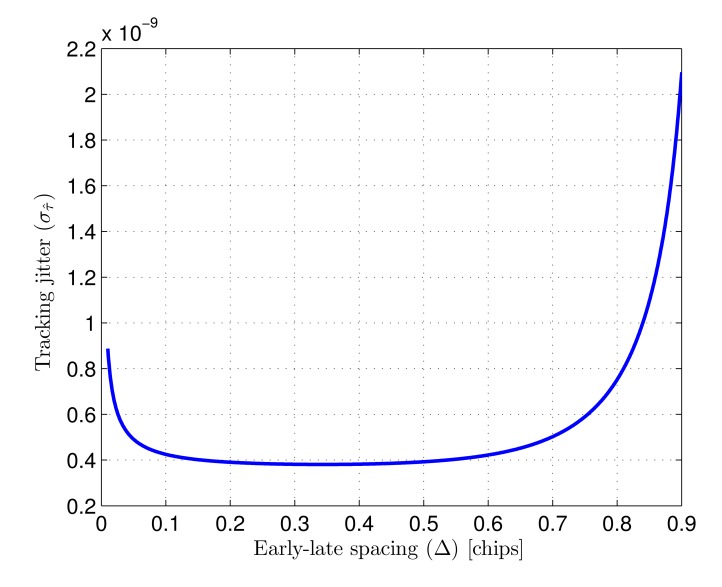
Typical tracking error (*σ*_τ̂_) caused by AWGN as a function of early-late spacing (Δ) (SNR=75 dBHz, *W_L_* = 100 kHz, *f_c_* = 25 MHz.).

Figure 5 shows the typical tracking error caused by noise as a function of Δ, calculated for a loop bandwidth *W_L_* of 100 kHz, a chip-rate *f_c_* of 25 MHz and a SNR of 75 dBHz, where SNR is defined as

(19) $$\text{SNR}=\frac{P_{r}}{N_{0}/2}$$

The tracking error in Figure 5 is only shown for values of Δ up to 0.9 chips since, as can be seen in Figure 4 for Δ = 1 chip, the discriminator slope at the origin becomes very small for higher values, critically increasing the resulting tracking jitter.

As can be seen in the figure, the early-late spacing that minimizes tracking jitter is approximately 0.35 chips. There is extensive literature on spread-spectrum ranging centered on GNSS receivers based on similar early-late processing. In this context it has been demonstrated that narrower correlation spacing yields higher multipath rejection [10,22–24]. Taking this into account, together with the tracking jitter analysis, an early-late spacing of 0.25 chips is selected and considered from now on in the performance study. This value provides a good noise behavior and is expected to yield higher multipath rejection than larger spacings while, being a simple fraction of a chip-time, presents little hardware complexity in the implementation of the corresponding delays.

The discriminator slope at the origin and the transfered noise power spectral density can now be calculated for the selected Δ = 0.25 chips, yielding an expected tracking variance

(20) $$\sigma_{\hat{\tau }}^{2}=T_{c}^{2}\frac{W_{L}}{64\text{SNR}}$$

### 4.2. Dynamic Error

The dynamic error sources affecting the tracking stage are the displacement of the target where the emitter is boarded and frequency errors due to the lack of synchronization between emitter and receivers. The total delay rate of change is modeled considering a worst case situation for both effects.

The maximum variation of the input delay caused by movement of the target is given by

(21) $$\Delta_{\tau }^{\text{target}}=\frac{V_{\text{target}}}{c}$$

where *c* is the propagation speed of the optical signal, approximated by the propagation speed in vacuum (≈3 x 10^8^ m/s) and *V*_target_ is the speed of a target in the direction towards the receiver.

The maximum variation of the input delay due to frequency errors, for a reference clock accuracy in ppm *∈*_CLK_, is

(22) $$\Delta_{\tau }^{\text{CLK}}=1-\frac{1}{1-2\in_{\text{CLK}}10^{-6}}$$

assuming frequency deviations of emitter and receiver reference clocks are of opposite sign and equal to the maximum deviation given by the manufacturer.

Therefore, the maximum delay rate of change for both effects considering the worst case in terms of sign of the variation is

(23) $$\Delta_{\tau }=\frac{V_{\text{target}}}{c}+1-\frac{1}{1-2\in_{\text{CLK}}10^{-6}}\approx \frac{V_{\text{target}}}{c}+2\in_{\text{CLK}}10^{-6}$$

In practical terms the dynamic error will be dominated by the clock errors. As an example considering some realistic case for both dynamic error sources: a target moving at 1 m/s directly towards one receiver would cause a delay variation of 3 ns/s. On the other hand, if the timing systems in emitter and receivers have deviations of 10 ppm in opposite directions, the apparent delay variation is 20 *μ*s/s; *i.e.*, the rate of change of the delay due to the lack of synchronism is nearly 4 orders of magnitude faster than the one caused by the movement of the target.

Note that the final range difference estimation, based on phase measurements over the despread sine signals as briefly explained in Section 2, is not affected by frequency errors. Those errors are common for all receivers, hence canceled in the subtraction between two estimated phases. Analyzing the phase measurement performance is not in the scope of this paper, however, it is noteworthy that the final estimation bandwidth of the ranging system will be adapted to the much slower dynamics set by the movement of the target. This implies the possibility of using reduced final bandwidths to increase noise absorption, hence achieving smaller noise-related errors than those yielded by the tracking stage.

The error of the loop in tracking a linear delay variation whose slope is Δ*_τ_* is used to define the dynamic error of the tracking stage. This error is estimated by analyzing the tracking error of the closed-loop transfer function to a ramp function whose slope is Δ*_τ_*.

In the most simple case, using a first order loop filter with 3 dB cut-off frequency *f_h_*, the closed-loop transfer function is

(24) $$H\left(s\right)=\frac{2\pi f_{h}K_{d}}{s+2\pi f_{h}\left(1+K_{d}\right)}$$

where *K_d_* is the discriminator gain defined in Equation (17).

The function defining the linear delay variation is

(25) $$x_{r}\left(t\right)=\Delta_{\tau }t$$

After the initial transient, when $t\gg \frac{1}{2\pi f_{h}\left(1+K_{d}\right)}$, the system output can be approximated as

(26) $$y_{r}\left(t\right)\approx \Delta_{\tau }\left(t-\frac{1}{2\pi f_{h}\left(1+K_{d}\right)}\right)$$

so the tracking error is

(27) $$\in_{\hat{\tau }}=x_{r}\left(t\right)-y_{r}\left(t\right)=\frac{\Delta_{\tau }}{2\pi f_{h}\left(1+K_{d}\right)}$$

To provide an easier comparison with the noise-related results, the tracking error can be rewritten considering the noise equivalent bandwidth of the first order loop

(28) $$W_{L}=\frac{\pi }{2}f_{h}\left(1+K_{d}\right)$$

yielding

(29) $$\in_{\hat{\tau }}=\frac{\Delta_{\tau }}{4W_{L}}$$

### 4.3. Total Error

The trade-off between noise-related error and dynamic error is linked by the loop bandwidth. The total error considering both sources, defined as the addition of the typical error (1*σ*) caused by noise and the dynamic error, is

(30) $$\in_{T}=\frac{T_{c}}{8}\sqrt{\frac{W_{L}}{\text{SNR}}}+\frac{\Delta_{\tau }}{4W_{L}}$$

The optimum bandwidth is the value *W_L_* which minimizes the total error function for a given chip-rate under certain SNR and delay rate of change. This optimum bandwidth is

(31) $$W_{L}=\sqrt[{3}]{\frac{16\Delta_{\tau }^{2}\text{SNR}}{T_{c}^{2}}}$$

**Figure 6.**
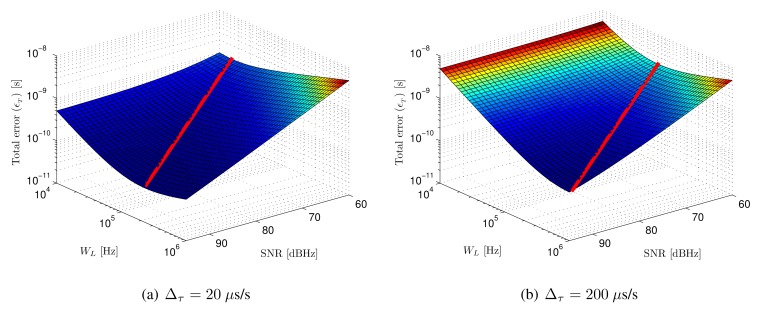
Total error and total minimum error (red line) as a function of input SNR and loop bandwidth (*W_L_*) for two different delay rates of change (Δ*_τ_*).

The total error as a function of input SNR and loop bandwidth is shown in Figure 6a,b. The minimum error for every SNR value is depicted using a red line. The error shown in Figure 6a has been computed for a delay variation of 20 *μ*s/s. This delay variation is dominated the by emitter-receiver lack of synchronism, calculated for a clock error difference of 20 ppm. Figure 6b shows the total error considering the master clocks of both systems would be 10 times less accurate, yielding a clock error difference of 200 ppm (*i.e.*, 200 *μ*s/s).

The dependence of total error with the input signal SNR and the selected loop bandwidth can be seen observing both figures. When the loop bandwidth is too small, the error increases due to the slow dynamic response of the system. On the other hand, when the loop bandwidth is too large, the error is dominated by noise, since a higher amount of noise spectra is integrated in the loop, providing very reduced noise filtering. The optimum value of the loop bandwidth for every SNR is higher when the delay rate of change is higher, since faster system dynamics are necessary to yield the same dynamic error.

**Figure 7.**
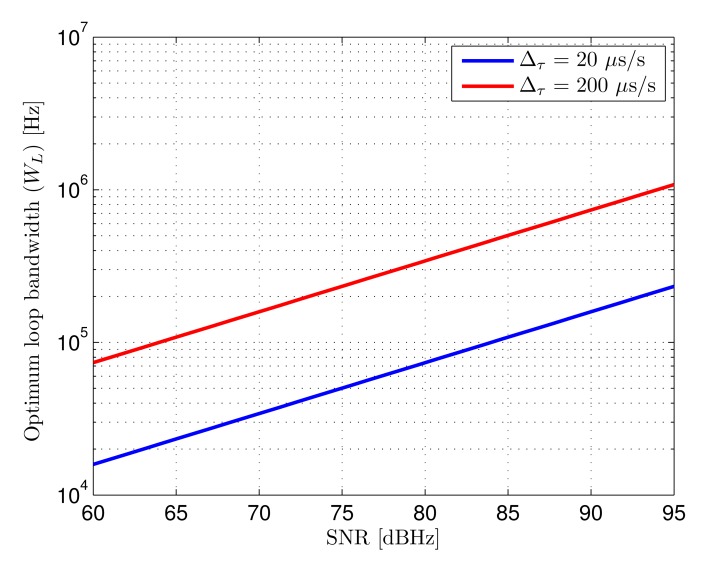
Loop bandwidth (*W_L_*) that minimizes the total error (*∈_T_*) as a function of input SNR for two different delay rates of change (Δ*_τ_*).

The loop bandwidths that minimize the total error for both delay rates of change as a function of SNR are shown in Figure 7. In practical terms, it would be generally convenient aiming at minimizing the maximum error for any possible case in a given scenario. This would mean selecting the optimum bandwidth for the worst case SNR taking into account the expected delay rate of change.

### 4.4. Multipath Behavior

When multipath components are present in the input signal of the tracking loop, the discriminator output is modified by those, causing the discriminator zero-crossing to have an unknown offset in relation to the point where the estimation error *τ*_LOS_ − *τ̂* is zero. The magnitude of this error depends on the particular delays and power relations of all the components forming the incoming signal. These delays and power relations depend on the position of the target and the particular environment geometry and properties, causing, therefore, an unknown multipath error.

The displacement of the zero-crossing point of the discriminator in the presence of one NLOS component is developed next to provide a preliminary estimation of the multipath error. A deep study on multipath, based in the following approach and including an indoor optical multipath model will be presented in a future contribution.

The received signal affected by one multipath component is

(32) $$s\left(t\right)=\sqrt{2P_{\text{LOS}}}m\left(t-\tau_{\text{LOS}}\right)+\sqrt{2P_{\text{MP}}}m\left(t-\tau_{\text{MP}}\right)$$

With this input signal, the output of the early and late correlators is formed by the addition of two versions of the expected signal autocorrelation for two different delays and powers. This correlations, applying an early-ate spacing of 0.25 chips, are

(33) $$R_{E}=\sqrt{2P_{\text{LOS}}}R_{m}\left(\tau_{\text{LOS}}-\hat{\tau }+\frac{T_{c}}{8}\right)+\sqrt{2P_{\text{MP}}}R_{m}\left(\tau_{\text{MP}}-\hat{\tau }+\frac{T_{c}}{8}\right)$$

(34) $$R_{L}=\sqrt{2P_{\text{LOS}}}R_{m}\left(\tau_{\text{LOS}}-\hat{\tau }-\frac{T_{c}}{8}\right)+\sqrt{2P_{\text{MP}}}R_{m}\left(\tau_{\text{MP}}-\hat{\tau }-\frac{T_{c}}{8}\right)$$

The discriminator output formed by this early and late correlations is

(35) $$\begin{array}{ll} D_{0.25}\left(t,\delta \right) & =\sqrt{2P_{\text{LOS}}}\left[R_{m}\left(\delta T_{c}+\frac{T_{c}}{8}\right)-R_{m}\left(\delta T_{c}-\frac{T_{c}}{8}\right)\right]\\ & +\sqrt{2P_{\text{MP}}}\left\{R_{m}\left[\left(\delta +\delta_{\text{MP}}\right)T_{c}+\frac{T_{c}}{8}\right]-R_{m}\left[\left(\delta +\delta_{\text{MP}}\right)T_{c}-\frac{T_{c}}{8}\right]\right\} \end{array}$$

where *δ* is the estimation error (*τ*_LOS_ − *τ̂*) and *δ*_MP_ is the difference between NLOS and LOS delays, both normalized to *T_c_*.

The normalized multipath error *∈*_MP_ for some given *P*_LOS_, *P*_MP_, and *δ*_MP_ is the value of *δ* in the linear region of the discriminator that makes

(36) $$D_{0.25}{\left(t,\delta \right)|}_{\delta =\in_{\text{MP}}}=0$$

**Figure 8.**
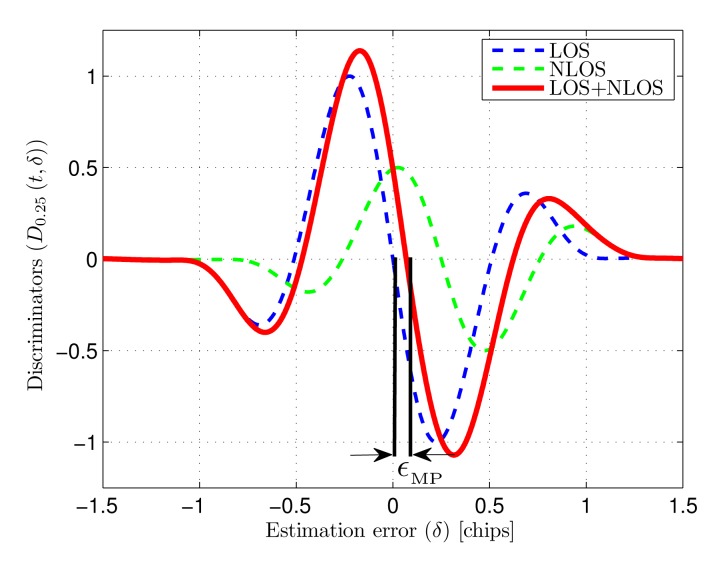
Discriminator functions associated to LOS, NLOS and composed signal (NLOS to LOS optical power ratio = 25%).

Figure 8 shows the discriminator functions associated to a LOS path and a NLOS path, together with the composed discriminator formed by both. The optical power ratio between NLOS and LOS is 25% and the NLOS to LOS delay is 10 ns (*δ*_MP_ = 0.25 chips at 25 MHz chip-rate). It can be seen how the zero-crossing point of the composed discriminator presents an offset compared to the multipath-free discriminator. This offset is the the normalized multipath error (*∈*_MP_) caused by the NLOS path.

## 5. Results

### 5.1. Test Set-Up and Scenario

In order to validate the theoretical analysis defined so far, the expected results have been compared with measurements carried out in a digital implementation (Simulink) of the defined ELDLL tracking architecture. In turn, input signals of the ELDLL containing the real effects to be tested (noise, delay variation and multipath) have been obtained in two different ways:

- Simulated IR link (synthetic signals): These signals are generated directly in the digital domain. The effect of the IR link bandwidth limitation is introduced by digital filtering whose frequency response is designed to emulate closely that of a first order analog low-pass filter. Wideband noise is generated with the aimed power spectral density and band-limited by digital filtering emulating the frequency response of the anti-aliasing filter that would precede the digitization.
- Emulated IR link (digitized analog signals): An emulation of the IR link with a wired connection has been implemented using a high speed arbitrary function generator and a 5 GS/s digitizer. The main difference between these signals and the all-digital synthetic ones is the lack of synchronism between both instruments. This implies a more realistic signal processing since the local replicas in the tracking stage are not frequency locked with the incoming signal. The effect of the IR bandwidth limitation is also introduced by digital filtering before the signal generation. The adequate noise density in every case is introduced by adding an external band-limited AWGN source in the analog channel before digitization. The dynamic tests are carried out using these signals by adding a linear phase modulation to the emitted signal.

**Figure 9.**
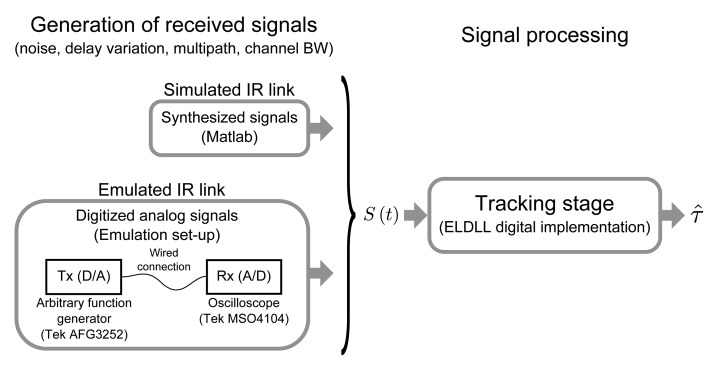
Diagram of the system validation procedure: Received signals generation (simulated and emulated IR link) and ELDLL digital implementation.

The second approach for generating the input signals yields a more realistic approximation, however, flexibility and resolution of these tests are poorer than in the synthetic approach, so both methods are used for validation. Both types of input signals are defined or digitized with a sampling frequency of 5 GS/s, which has been tested to be high enough so that the effects of the digitization can be neglected in relation to the effect of noise and dynamic errors analyzed with the continuous-time approach usually considered in the literature. Figure 9 shows a diagram of the complete validation set-up.

**Figure 10.**
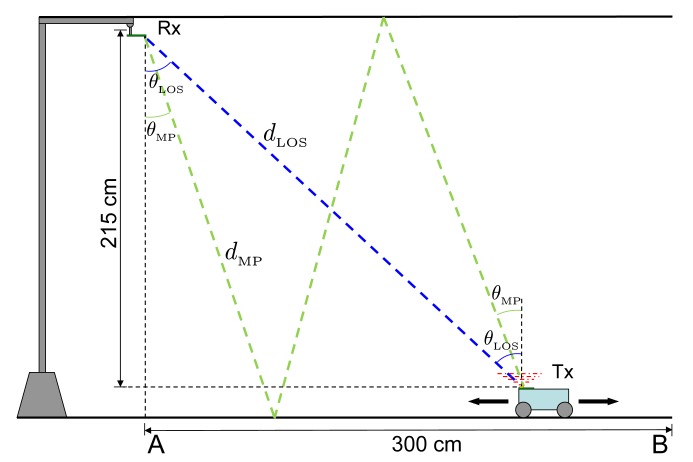
Emitter-receiver IR link defined for the results. LOS propagation path (blue) and one NLOS propagation path (green) due to ceiling-floor reflection.

The scenario defined for the results, *i.e.*, the values of the parameters used to generate the input signals and to compute the theoretical expressions, are based on the IR emitter-receiver link shown in Figure 10. The figure represents a practical link configuration in a standard indoor environment, where both emitter and receivers boresights are parallel. The possible target positions are those between points A and B in the figure, being the positions of maximum and minimum received power respectively. Two propagation paths are depicted: a LOS path, defined by its length *d*_LOS_ and emission-reception angle to the vertical *θ*_LOS_, and a NLOS path assuming a specular ceiling-floor reflection, defined by its length *d*_MP_ and emission-reception angle *θ*_MP_.

The received optical power *P_o_*, assuming a Lambertian emitter, is given by

(37) $$P_{o}=I_{e}\frac{A_{s}}{d^{2}}\cos^{2}\left(\theta \right)$$

with *I_e_* being the radiant intensity in the normal direction to the emitter surface, *A_s_* being the sensible area of the photodetector, *d* being the propagation path length, and *θ* being the emission and reception angle to the vertical.

The received electrical power *P_r_* at the output of the receiver low-level conditioning stage that transforms the generated photocurrent into a voltage is given by

(38) $$P_{r}=P_{o}RG_{f}G_{\upsilon }$$

with ℛ being the responsivity of the photodetector at the peak emission wavelength (870 nm;, *G_f_* being the gain of the transimpedance amplifier and *G_υ_* being the voltage gain before digitization, both considered flat in the link bandwidth.

The SNR of the received signal as a function of *P_r_* and the noise spectral density *N*_0_/2 at the output of the receiver conditioning stage was defined in Equation (19).

Table 1 shows the device parameters used in the numerical and experimental analysis, which correspond to a practical selection of low-cost up-to-date IR-LED and Si-PIN photodiode to implement a 870 nm link with 25 MHz chip-rate using simple optics on both devices.

Considering these parameters, the received optical power when the target is placed in the extreme points B and A of Figure 10 are 7.5 nW and 65 nW respectively. These yield an electrical power *P_r_* in the output of the conditioning stage of 46 *μ*W and 3.4 mW. Taking into account the noise spectral density at this point, the possible SNR values in the defined scenario range from 65.5 dBHz to 84 dBHz.

### 5.2. Experimental Results

The results shown in this section correspond to the main error sources analyzed in this work, *i.e.*, tracking error caused by AWGN and dynamic error, together with some preliminary results of the expected multipath error in the defined scenario.

**Figure 11.**
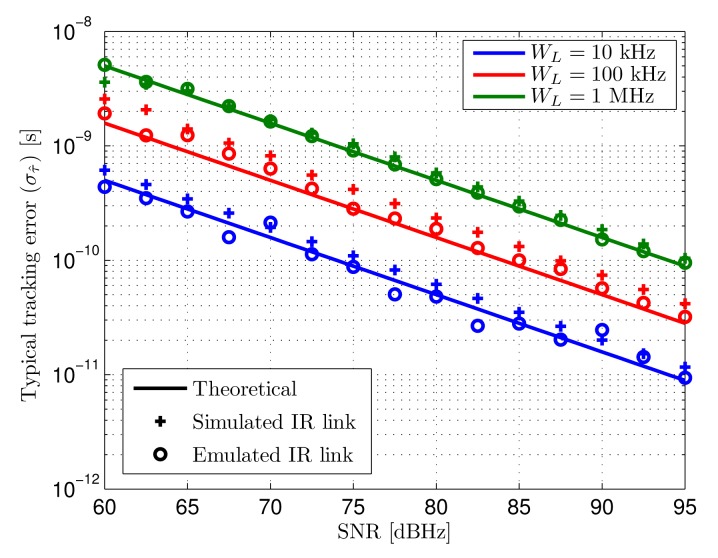
Typical tracking error (*σ_τ̂_*) caused by AWGN as a function of input SNR for different loop bandwidths (*W_L_*). Theoretical (solid), input signal from simulated IR-link (crosses) and input signal from emulated IR-link (circles).

**Tracking jitter**: Figure 11 shows the standard deviation of the tracking error caused by AWGN. The theoretical results and tests have been obtained for a SNR range than includes the values calculated in the defined scenario. Three different loop bandwidths has been used, selected as practical values considering the optimization between noise and dynamic errors addressed in Section 4.3.

Solid lines represent the theoretical estimated tracking error, while crosses and circles are the measured tracking error in the output of the tracking loop when the input signals are those explained above, being synthetic and digitized analog signals respectively.

The measured tracking errors both for synthetic and digitized analog inputs are very similar to the expected results. Higher differences can be observed for low loop bandwidths since the noise related error in those cases is smaller. In these situations, other error sources such as the quantization effects of digitization and the time-discretization of the signals start showing a non-negligible effect compared to the noise-related source. Note that the term “simulated” does not refer to computations of a model of the system but to the signals introduced in the loop implementation being generated directly in the digital domain, based on a signal model that includes the main real effects to be evaluated.

The expected typical tracking error caused by noise in the defined scenario for a 10 kHz loop bandwidth goes from 300 ps to 30 ps between the minimum and maximum SNR positions B and A of Figure 10. When higher loop bandwidths are selected to minimize total error due to more demanding dynamics (higher asynchronism), the achieved precision goes from 900 ps to 90 ps for 100 kHz bandwidth and 3 ns to 300 ps for 1 MHz bandwidth.

**Figure 12.**
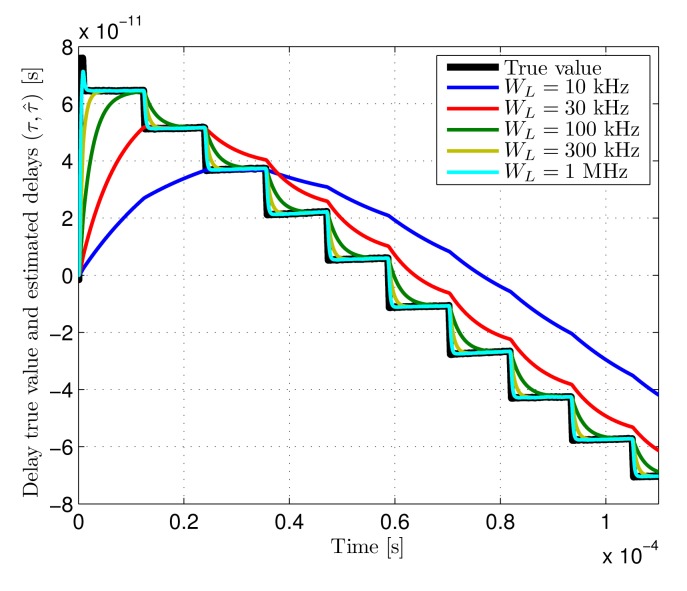
Delay true value (*τ*) and measured delays (*τ̂*) with different loop bandwidths (*W_L_*) for a delay rate of change (Δ*_τ_*) of 1 *μ*s/s. True-value delay steps (due to test set-up HW limitations) ≈18 ps.

**Dynamic error:** the dynamic error results used to validate the theoretical expected values are shown next. The dynamic error is measured indirectly due to hardware limitations of the test set-up. The phase modulation of the function generator used for the IR link emulation does not allow generating a linear delay variation on the signals introduced in the processing architecture. Instead, delay steps of approximately 18 ps are introduced in the emitted signal with the adequate periods so that the average delay variations are those defined for the tests. The output of the system is obtained for this input signal with different loop filters. Since the closed-loop frequency response of the system is known to be of first order, the input and output signals are used to carry out a system identification in order to calculate the closed-loop cut-off frequency of the system. Finally, the identified value for every loop filter is used to calculate the tracking error applying Equation (27), where the identified value would correspond to the theoretical closed-loop cut-off frequency *f_h_* (1 + *K_d_*). Figure 12 shows the input signal delay true value and the system outputs for different loop bandwidths when the introduced average delay rate of change is 1 *μ*s/s.

**Figure 13.**
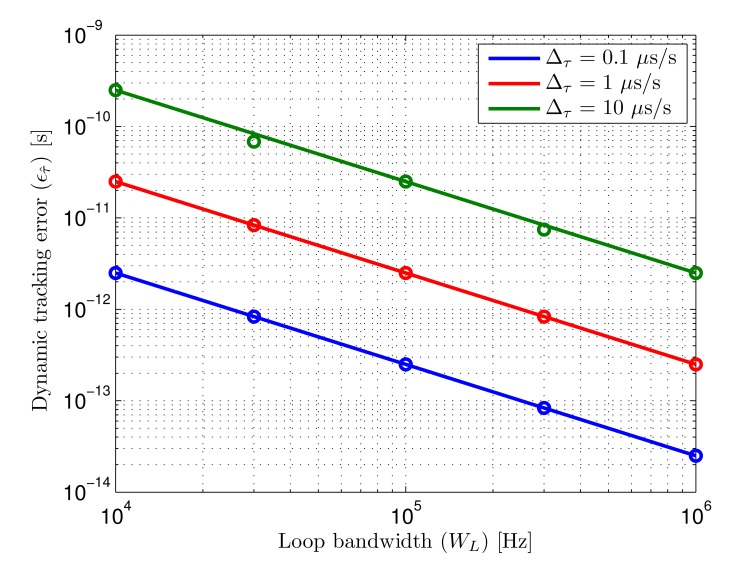
Dynamic error as a function of loop bandwidth (*W_L_*) for three delay rates of change (Δ*_τ_*). Theoretical (solid) and IR link analog emulation (circles).

Figure 13 shows the dynamic error as a function of loop bandwidth for two different delay rates of change. The theoretical results are shown in solid lines while the measured error for the bandwidths depicted in Figure 12 are shown in circles.

The dynamic errors, measured indirectly by system identification of the known closed-loop cut-off frequency, agree with the theoretical expected results for the test values of Δ*_τ_*. Slight differences can be observed for the highest rate of change, caused by higher inaccuracies in the faster phase modulation of the generated signals.

**Figure 14.**
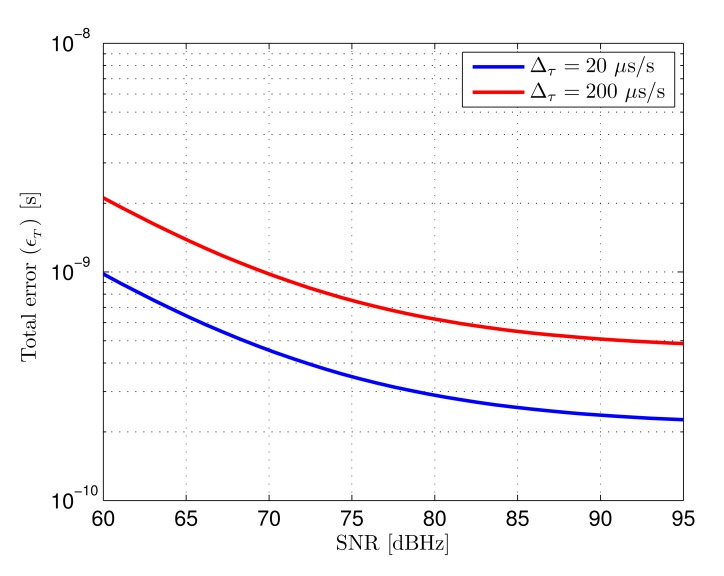
Expected total error (*∈_T_*) as a function of input SNR for two delay rates of change (Δ*_τ_*).

**Total error:** the total error of the tracking loop considering the combined effect of AWGN and dynamic error is given by the addition of both contributions, defined in Equation (30), when the optimum bandwidth is selected for the expected delay variation. Considering the SNR range in the defined scenario, going from 65.5 dBHz to 84 dBHz, if the expected delay variation is 20 *μ*s/s, the loop bandwidth selected for the minimum SNR level would be approximately 24 kHz, as can be seen in Figure 7. In this case the error goes from 640 ps to 250 ps. If the expected delay variation is 200 *μ*s/s, the selected bandwidth would be approximately 110 kHz, yielding an error between 1.4 ns and 550 ps. The theoretical total error as a function of SNR for both delay rates of change is shown in Figure 14. It can be seen that when the signal quality improves, the total error stops decreasing with SNR since it becomes dominated by dynamic effects.

The goal of the tracking stage is providing a relative delay estimation to generate the local replicas with the adequate phase for the subsequent DSSS demodulation. The coherence of the demodulation depends on the alignment between the involved signals. The total errors expressed in relative chip time, for the 25 MHz chip-rate used for the results, would be between 1.6% and 0.6% of a chip time for Δ*_τ_* = 20 *μ*s/s, and between 3.5% and 1.4% of a chip time for Δ*_τ_* = 200 *μ*s/s.

**Figure 15.**
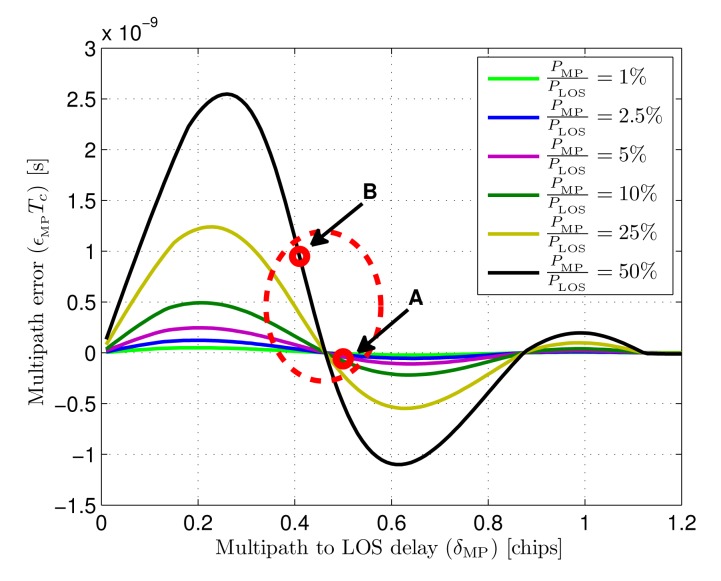
Multipath error (*∈*_MP_*T_c_*) as a function of the delay between LOS and NLOS components for different optical power ratios (*f_c_* = 25 MHz).

**Multipath behavior:** the multipath error has been calculated by measuring the displacement of the zero crossing point over simulations of the discriminator affected by one NLOS component Equation (35). The optical power of the multipath component has been set to different values between 1% and 50% of the LOS component power, sweeping delays (*τ*_MP_ − *τ*_LOS_) from 0.01 to 1.2 chips. The resulting errors are shown in Figure 15. Observing the results, it can be seen that maximum multipath errors are caused by multipath to LOS delays around 0.25 chips, moving closer to 0.3 chips for stronger multipath components. The maxium error value, for a multipath optical power of 50% the LOS power, is a 6.5% of a chip duration, approximately 2.6 ns for a chip-rate of 25 MHz.

To provide a preliminary idea of the magnitude of the multipath error in a practical case, two multipath situations have been defined associated to the test scenario shown in Figure 10. The NLOS path reaching the receiver after a double reflection in ceiling and floor, assuming specular reflection in both surfaces, has been calculated for points A and B in the figure. Table 2 show the associated delays and received optical power ratios for the LOS and NLOS components in both points, together with the estimated multipath error extracted from the simulations shown in Figure 15.

As can be seen in the results for the given scenario, when the target is directly under the receiver in position A, the doubly-reflected multipath would introduce an error of approximately 0.2% of a chip duration. On the other hand, when the target is in position B, the furthest from the receiver, the multipath error would be approximately a 2.4% of a chip duration. The multipath error corresponding to both positions is depicted in Figure 15, together with a slashed red ellipse representing the approximated working region, in terms of multipath error, for the practical case used in the results.

This is a promising result considering that 0% tracking error means that 100% of the LOS power is recovered in the subsequent DSSS demodulation, and that a 100% error means no power recovery. Taking into account the overall error contributions apart from multipath, a total tracking error of 6% of a chip time is achieved in the worst case (position B). Assuming a linear relationship between recovered power and alignment error in the DSSS demodulation, which is a valid approximation at this point [20], this error would mean that 94% of the LOS power is recovered for the phase estimation while only a 65% of the NLOS power is despread. This would mean a multipath interference power reduction of 31% in position B, while, in the less severe conditions of position A, the multipath interference would be reduced by 51%.

With these results, the final ranging accuracy after the phasemeter, considering noise and system dynamics, can be predicted. An estimation of the multipath error in the range estimation requires a deeper study that will be presented in future contributions. The final estimation bandwidth will be adapted to the dynamics of the target displacement since, after the phasemeter, frequency errors are canceled by differential measurements. This allows reducing the output bandwidth to the order of tens of Hz, providing much higher noise absorption than in the tracking stage. Assuming a final bandwidth of 30 Hz, adequate for tracking targets moving at 1 m/s [5], the noise-related error standard deviation, obtained as the inverse of the SNR square root, which is a widely used approximation in phase-based estimation, would be 5.7 mm. On the other hand, the dynamic error for a target moving at 1 m/s, calculated as the error of a first order filter tracking a linear range variation, would be 7.5 mm.

This work, although proposing a method for optical ranging, it is closely linked with RF technology, since the tracking stage designed and analyzed in this paper is based on a delay tracking method typically used in RF ranging and communications. Due to this, aiming at providing a comprehensive idea of the proposed system performance and possibilities in a more general context, it is compared next with the main RF-based ranging technologies that are suitable for the application field of this work, i.e., indoor positioning. Table 3 shows a comparison between these systems and the proposed IR-based system. The typical measuring principles in which every technology is based are provided. The comparison includes methods based on Time of Arrival, whose particular technique to estimate this time is specified in the table, together with other less accurate RF-ranging approaches. Accuracy refers to typical accuracy of up-to-date research or market products of the corresponding technology. Range refers to single nodes of the particular technology. Cost only aims at providing comparative information between the different alternatives, while installation refers to the requirement of a fixed set-up in the localization environment. The information in the table has been extracted from recent surveys on indoor positioning technological alternatives [27,28].

The most comparable RF-based alternatives to the system proposed in this work are those based on ToA. The other alternatives (WLAN, RFID…) are based on some kind of power-based or proximity-based estimation. These systems provide less accurate positioning but also lower costs, since installation is either non-required because of using infrastructure already deployed for communications (WLAN, GSM…), or rather simple like in most RFID options where only low-cost self-powered tags have to be deployed. ToA systems are based on the estimation of the propagation delay of the received signals. The way this delay is estimated differs between methods: UWB systems usually make use of their very high time-resolution to make direct ToA estimations of very short pulses, which allows high accuracy and a good discrimination of multipath components. HS-GNSS use high performance receivers (integration over multiple intervals) and signal processing (additional data link, high resolution parallel correlations) to access GNSS signals in environments where they are too weak to be processed with standard receivers. Although they can provide position information in environments that are usually considered GNSS-denied, accuracy is still poor due to low signal levels and strong multipath interferences. Pseudolites systems are inspired in GNSS, sharing similar signal structure that allows correlation-based ToA estimations. The emitters, instead of satellites, are static antennas deployed in the application environment, allowing much higher received power hence precision.

As can be seen in the table, the proposed system slightly surpasses the best accuracy results of pseudolites and UWB, although its achievable coverage is lower. This is due to the required LOS between emitter and receiver in the optical system, which, though recommended to avoid severe signal attenuation, is not completely necessary in the RF alternatives. On the other hand, in reduced environments where high signal levels can be guaranteed and multipath is not severe, the higher stability of the optical link against environmental conditions allows achieving accuracies in the mm level, making the proposed system an adequate alternative for applications such as mobile robotics or specific manufacturing control that require accurate dynamic positioning in not very large indoor environments or clearly defined areas.

## 6. Conclusions

A tracking stage for spread-spectrum optical signals based on an early-late delay-locked loop was defined and studied in this work. The use of spread-spectrum tracking techniques is typical in RF-based ranging and communications. However, its application to indoor optical ranging, constrained to different bandwidths and signal structures due to the specific features of indoor localization, is a novel approach that requires a particularized analysis.

The expressions of the tracking error caused by AWGN and varying delays in the input signal are derived and used to optimize the design parameters of the tracking loop. The analysis concludes that an early-late spacing of 0.25 chips represents an adequate value to minimize the effect of noise while also providing a good multipath behavior and low HW implementation complexity. The selection of an adequate loop bandwidth has also been addressed, aiming at minimizing the total error by balancing noise-related and dynamic-related errors. An expression of the optimum loop bandwidth as a function of the input signal SNR and expected delay rate of change is provided.

The results for the selected design parameters are calculated for a realistic indoor scenario defined for the tests considering a practicable IR link built with a low-cost up-to-date IR-LED and Si-PiN photodiode. The expected SNR range in this scenario for all possible target positions goes from 65.5 dBHz to 84 dBHz. The worst-case total typical error of the delay estimated by the tracking stage is 1.6% of a chip time when the clock error difference between emitter and receiver is 20 ppm, and 3.5% of a chip time when the clock error is 200 ppm, both calculated for a chip-rate of 25 MHz.

Multipath errors are the main accuracy-limiting factor in wide-coverage optical telemetry with the aforementioned application. The behavior of the tracking stage studied in a simple but realistic scenario has been addressed, determining the multipath error bounds in a practical locating configuration. Preliminary results, showing multipath errors below 2.5% of a chip time, allow a positive expectation on the multipath-related performance of the whole system, while the analytical approach serves as a link to near future works on the DSSS demodulation stage and its application to a complete multipath model in a real environment.

The achieved results demonstrate that the tracking stage of the proposed system can provide an accurate delay estimation of the received optical signal. The estimation accuracy is adequate to generate the local replicas of the expected sequence to be used for coherent demodulation prior the phase-based range estimation. Considering the worst case situation in terms of SNR, delay variation and multipath, a total tracking error of 6% of a chip time is achieved. This is a promising result for the whole ranging system performance. In terms of noise and dynamic behavior, a total error below 1.5 cm can be expected under the given conditions and tracking performance. Providing a quantitative value for the multipath error in the range estimation requires a deeper study that will be presented in future contributions, however, a minimum 30% of NLOS-to-LOS power reduction can be expected even under unfavorable conditions.

## Figures and Tables

**Table 1.** Emitter (IR-LED: Hamamatsu L12170), receiver (Si-PIN photodiode: OSI BPX-65R) and conditioning stage parameters to implement an IR link for 25 MHz chip-rate.

	**Parameter**	**Value**	**Units**
Emitter	Radiant intensity (*I_e_*)	36	mW/sr
	Peak emission wavelength (*λ_p_*)	870	nm
	Half-intensity angle (*α*_1/2_)	60	*o*
	Cut-off frequency (*f_cTx_*)	40	MHz

Receiver	Sensible area (*A_s_*)	4	mm^2^
	Responsivity @ 870 nm (ℛ)	0.57	A/W
	Cut-off frequency (*f_cRx_*)	160	MHz
	Transimpedance gain (*G_f_*)	33	kΩ
	Voltage gain (*G_υ_*)	100	V/V
	Noise spectral density (*N*_0_/2)	1.34 × 10 ^−11^	W/Hz

**Table 2.** Delays and optical power ratios associated to LOS and NLOS paths in point A and B of Figure 10.

**Optical Power Ratio** $\left(\frac{P_{\text{MP}}}{P_{\text{LOS}}}\right)$		**Delay Difference** (*δ*_MP_)	**Multipath Error** (*∊*_MP_*T*_c_)
Position A	7%	20 ns (0.5 chips)	60 ps (0.002 chips)
Position B	47%	16.6 ns (0.41 chips)	950 ps (0.024 chips)

**Table 3.** Comparison of the IR-based ranging proposal with different RF-based ranging technologies which are typically applied in indoor localization.

**Technology**	**Measuring Principle**	**Accuracy**	**Range [m]**	**Cost (Installation)**
UWB^1^	ToA ^5^ (impulse radio / PRN phase)	cm-m	1–50	Medium/High (y)
WLAN ^2^	Fingerprinting / RSSI ^6^	m	20–50	Low (n)
RFID ^3^	Fingerprinting / CoO ^7^	dm-m	1–50	Medium (y)
Other RF	Fingerprinting / CoO ^7^ / RSSI ^6^	m	10–1000	Low/Medium (y/n)
HS-GNSS ^4^	ToA ^5^ (PRN and carrier phase)	m	“Global”	Medium/High (n)
Pseudolites	ToA ^5^ (PRN and carrier phase)	cm-dm	10–1000	High (y)
IR proposal	ToA ^5^ (PRN and sine phase)	mm-dm	1–10	Medium/High (y)

1 Ultrawideband;

2 Wireless Local Area Network;

3 Radio Frequency Identification;

4 High-Sensitivity Global Navigation Satellite System;

5 Time of Arrival;

6 Received Signal Strength Indicator;

7 Cell of Origin.

## References

[1] Gu Y., Lo A., Niemegeers I. (2009). A survey of indoor positioning systems for wireless personal networks. Commun. Surv. Tutor..

[2] Mautz R., Tilch S. Survey of optical indoor positioning systems.

[3] Dragunas K. Indoor multipath mitigation.

[4] Moision B., Erkmen B.I. Achievable precision for optical ranging systems.

[5] Martín Gorostiza E., Lázaro Galilea J.L., Meca Meca F.J., Salido Monzú D., Espinosa Zapata F., Pallarés Puerto L. (2011). Infrared sensor system for mobile-robot positioning in intelligent spaces. Sensors.

[6] Braasch M.S. Performance comparison of multipath mitigating receiver architectures.

[7] Chen X., Dovis F., Peng S., Morton Y. (2013). Comparative studies of GPS multipath mitigation methods performance. IEEE Trans. Aerosp. Electron. Syst..

[8] Van Nee R.D.J. The Multipath Estimating Delay Lock Loop.

[9] Sanchez-Fernandez M., Aguilera-Forero M., Garcia-Armada A. (2007). Performance analysis and parameter optimization of DLL and MEDLL in fading multipath environments for next generation navigation receivers. IEEE Trans. Consum. Electron..

[10] Van Dierendonck A.J., Fenton P., Ford T. (1992). Theory and performance of narrow correlator spacing in a GPS receiver. Navigation.

[11] Veitsel V.A., Zhdanov A.V., Zhodzishsky M.I. (1998). The mitigation of multipath errors by strobe correlators in GPS/GLONASS receivers. GPS Solut..

[12] Kanekal S.M., Braasch M.S. Multipath mitigation with gated signal technology.

[13] McGraw G.A., Braasch M.S.

[14] Lay O.P., Dubovitsky S., Shaddock D.A., Ware B. (2007). Coherent range-gated laser displacement metrology with compact optical head. Opt. Lett..

[15] Shaddock D.A. (2007). Digitally enhanced heterodyne interferometry. Opt. Lett..

[16] De Vine G., Rabeling D.S., Slagmolen B.J., Lam T.T., Chua S., Wuchenich D.M., Shaddock D.A. (2009). Picometer level displacement metrology with digitally enhanced heterodyne interferometry. Opt. Express.

[17] Segers L., Tiete J., Braeken A., Touhafi A. (2014). Ultrasonic multiple-access ranging system using spread spectrum and MEMS technology for indoor localization. Sensors.

[18] Esteban Delgado J.J., Marín García A.F., Bykov I., Heinzel G., Danzmann K. Free-space laser ranging and data communication.

[19] Kwak J.S., Lee J.H. (2004). Infrared transmission for intervehicle ranging and vehicle-to-roadside communication systems using spread-spectrum technique. IEEE Trans. Intell. Transp. Syst..

[20] Salido-Monzú D., Wieser A., Martín-Gorostiza E., Lázaro-Galilea J.L., Domingo-Pérez F. Multipath mitigation for a phase-based infrared ranging system applied to indoor positioning.

[21] Betz J.W. The Offset Carrier Modulation for GPS Modernization.

[22] Van Dierendonck A.J., Braasch M. Evaluation of GNSS receiver correlation processing techniques for multipath and noise mitigation.

[23] Fenton P.C., Falkenberg B., Ford T.J., Ng K.K., van Dierendonck A.J. NovAtele's GPS receiver—The high performance OEM sensor of the future.

[24] Parkinson B.W., Spilker J.J., Axelrad P., Enge P. (1996). Multipath Effects. Global Positioning System. Theory and Applications.

[25] Peterson R.L., Ziemer R.E., Borth D.E. (1995). Code Tracking Loops. Introduction to Spread Spectrum Communications.

[26] Parkinson B.W., Spilker J.J., Axelrad P., Enge P. (1996). Fundamentals of Signal Tracking Theory. Global Positioning System. Theory and Applications.

[27] Mautz R. (2012). Indoor Positioning Technologies.

[28] Koyuncu H., Yang S.H. (2010). A survey of indoor positioning and object locating systems. Int. J. Comput. Sci. Netw. Secur.

